# Association of an Active Choice Intervention in the Electronic Health Record Directed to Medical Assistants With Clinician Ordering and Patient Completion of Breast and Colorectal Cancer Screening Tests

**DOI:** 10.1001/jamanetworkopen.2019.15619

**Published:** 2019-11-15

**Authors:** Esther Y. Hsiang, Shivan J. Mehta, Dylan S. Small, Charles A. L. Rareshide, Christopher K. Snider, Susan C. Day, Mitesh S. Patel

**Affiliations:** 1 Department of Medicine, University of California, San Francisco; 2 Perelman School of Medicine, University of Pennsylvania, Philadelphia; 3 Wharton School, University of Pennsylvania, Philadelphia; 4 Penn Medicine Nudge Unit, University of Pennsylvania, Philadelphia; 5 Department of Medicine, Crescenz Veterans Affairs Medical Center, Philadelphia, Pennsylvania

## Abstract

**Importance:**

Early cancer detection can lead to improved outcomes, but cancer screening tests are often underused.

**Objective:**

To evaluate the association of an active choice intervention in the electronic health record directed to medical assistants with changes in clinician ordering and patient completion of breast and colorectal cancer screening tests.

**Design, Setting, and Participants:**

A retrospective quality improvement study was conducted among 69 916 patients eligible for breast or colorectal cancer screening at 25 primary care practices at the University of Pennsylvania Health System between September 1, 2014, and August 31, 2017. Data analysis was conducted from January 21 to July 8, 2019.

**Interventions:**

From 2016 to 2017, 3 primary care practices at the University of Pennsylvania Health System implemented an active choice intervention in the electronic health record that prompted medical assistants to inform patients about cancer screening during check-in and template orders for clinicians to review during the visit.

**Main Outcomes and Measures:**

The primary outcome was clinician ordering of cancer screening tests. The secondary outcome was patient completion of cancer screening tests within 1 year of the primary care visit.

**Results:**

The sample eligible for breast cancer screening comprised 26 269 women with a mean (SD) age of 60.4 (6.9) years; 15 873 (60.4%) were white and 7715 (29.4%) were black. The sample eligible for colorectal cancer screening comprised 43 647 patients with a mean (SD) age of 59.4 (7.5) years; 24 416 (55.9%) were women, 19 231 (44.1%) were men, 29 029 (66.5%) were white, and 9589 (22.0%) were black. For breast cancer screening, the intervention was associated with a significant increase in clinician ordering of tests (22.2 percentage points; 95% CI, 17.2-27.6 percentage points; *P* < .001) but no change in patient completion (0.1 percentage points; 95% CI, −4.0 to 4.3 percentage points; *P* = .45). For colorectal cancer screening, the intervention was associated with a significant increase in clinician ordering of tests (13.7 percentage points; 95% CI, 8.0-18.9 percentage points; *P* < .001) but no change in patient completion (1.0 percentage points; 95% CI, −3.2 to 4.6 percentage points; *P* = .36).

**Conclusions and Relevance:**

An active choice intervention in the electronic health record directed to medical assistants was associated with a significant increase in clinician ordering of breast and colorectal cancer screening tests. However, it was not associated with a significant change in patient completion of either cancer screening test during a 1-year follow-up.

## Key Points

**Question:**

Is an active choice intervention in the electronic health record directed to medical assistants during primary care visits associated with improved rates of breast and colorectal cancer screening?

**Finding:**

In this quality improvement study of 25 primary care practices and 69 916 patients, the active choice intervention was associated with a significant increase in clinician ordering of both breast and colorectal cancer screening tests over time when compared with a control group of practices. However, the intervention was not associated with a significant change in patient completion of either type of cancer screening test during a 1-year follow-up.

**Meaning:**

Nudges facilitated by the electronic health record can increase clinician ordering of cancer screening tests but may need to be combined with other interventions to improve patient completion.

## Teaser

This quality improvement study evaluates the association of an active choice intervention in the electronic health record directed to medical assistants with changes in ordering and patient completion of breast and colorectal cancer screening tests.

## Introduction

Cancer is a leading cause of mortality in the United States.^1^ Although appropriate cancer screening can help to identify cancer at an earlier stage, it is often underused, which may lead to preventable deaths.^2,3,4,5,6^ The US Preventive Services Task Force guidelines recommend that eligible patients should be offered breast and colorectal cancer screening during primary care visits.^7,8^ However, the Centers for Disease Control and Prevention estimates that approximately 37% of adults have not been screened for colorectal cancer and 28% of women have not been screened for breast cancer.^9^

Nudges are subtle changes to choice architecture that can have important influences on medical decision-making.^10^ For example, prior work found that using active choice, a method that requires clinicians to accept or decline an order for breast or colorectal cancer screening tests in the electronic health record (EHR), significantly increased the rates of ordering cancer screening tests at 1 primary care practice compared with a control group of practices.^11^ This method has also been used to increase influenza vaccination and statin prescribing.^12,13^ However, this type of approach could lead to clinician alert fatigue.^14,15,16^ Therefore, before expanding to 3 other practices at our institution, the active choice intervention was redirected to medical assistants who could inform patients about eligibility for cancer screening, and template orders were made in the EHR for clinicians to review. The objective of this study was to evaluate the association of the active choice intervention with clinician ordering and patient completion of breast and colorectal cancer screening tests.

## Methods

The University of Pennsylvania Institutional Review Board approved this study and waived informed consent because it was infeasible given the retrospective study design and because the study posed minimal risk. This study followed the Standards for Quality Improvement Reporting Excellence (SQUIRE 2.0) reporting guidelines.

### Setting and Participants

The sample comprised adult patient visits from 25 primary care practice sites at the University of Pennsylvania Health System during the 3-year study period from September 1, 2014, to August 31, 2017, which included 2 years before and 1 year after the implementation of the EHR intervention. These practice sites were located in Pennsylvania and New Jersey, included both internal medicine and family medicine clinicians, and had at least 100 patients due for breast and colorectal cancer screening in each of the 3 years. Patients were included if they had at least 1 new or return clinic visit with their primary care physician (PCP) (attending or resident physicians) during the study period and they were due for either breast or colorectal cancer screening based on the US Preventive Services Task Force guidelines.^7,8^ For breast cancer screening, this included women between the ages of 50 and 74 years. For colorectal cancer screening, this included adults between the ages of 50 and 75 years. Using health maintenance information and data in the EHR, we looked back up to 10 years to evaluate prior patient interactions and screening tests to determine eligibility. Similar to prior work,^17,18^ we excluded patients if they were not due for cancer screening or changed PCPs at any time during the study period.

### Data

Similar to prior work,^11,18^ Clarity, an EPIC reporting database, was used to obtain data on patients, clinic visits, and cancer screening tests. Data on patients included demographic characteristics, insurance, comorbidities, PCP, and presence of cancer screening test results. Data on clinic visits included date, practice site, visit type, and presence of an order for cancer screening tests. Breast cancer screening could be completed by mammography. Colorectal cancer screening could be completed by colonoscopy, sigmoidoscopy, fecal immunochemical test, fecal occult blood test, or multitargeted stool DNA test. Electronic health record codes used to classify screening tests have been previously published.^18^ Household income level was obtained using US Census data on median household income based on zip code. Health insurance claims data were not available for this study.

### Intervention

Prior to the intervention, PCPs had to remember to manually check the EHR to determine whether a patient was due for cancer screening, discuss it with the patient, and then place an order for it in the EHR. From September 1, 2016, to August 31, 2017, 3 University of Pennsylvania Health System primary care practices implemented an active choice intervention in the EHR using a best-practice alert in EPIC directed to medical assistants. Prior to meeting with the clinician, patients met with a medical assistant to check their vital signs and prepare for the visit. At that time, the EHR checked for patient eligibility for breast and colorectal cancer screening and prompted medical assistants to accept or cancel an order for it. If accepted, the order would be templated (a pending order is made for the clinician to review and sign during the patient visit). This intervention was similar in design to prior work for influenza vaccination.^17^

### Main Outcome Measures

The primary outcome was clinician ordering of the screening test during the primary care visit. The secondary outcome was patient completion of a screening test (not necessarily linked to the order from the visit) within 1 year of the primary care visit.

### Statistical Analysis

To evaluate the association of changes in cancer screening rates with the active choice intervention, we used a difference-in-differences approach.^19,20^ Similar to prior work,^11,13,17^ we compared changes in cancer screening at the intervention vs control practices during the postintervention year relative to the 2 preintervention years. We used the patient as the unit of analysis and included all clinic visits during each year.

In the adjusted analysis, we used the SAS procedure PROC GENMOD to fit the model based on generalized estimating equations with a logit link and an independence correlation structure using PCP as the clustering unit.^21^ The model was adjusted for patient demographics (age, sex, race/ethnicity, and household income), insurance, Charlson Comorbidity Index,^22^ practice group (intervention or control site), clinic visit type (new or return), fixed effects by practice site, year, and calendar month. To obtain a difference-in-differences, we used an interaction term for practice group (intervention vs control) and year 3. To obtain the adjusted difference-in-differences in the percentage of patients along with 95% CIs, we used the bootstrap procedure, resampling patients 1000 times.^23,24^ Resampling of patients was done by PCP variable to maintain clustering at the PCP level. A test of controls was performed to test the null hypothesis of parallel trends between the intervention and control practices during the 2 preintervention years.^25^ Two-sided hypothesis tests used a significance level of *P* = .05; all analyses were conducted in SAS, version 9.4 (SAS Institute Inc).

## Results

The sample eligible for breast cancer screening comprised 26 269 women with a mean (SD) age of 60.4 (6.9) years; 15 873 (60.4%) were white, and 7715 (29.4%) were black (Table 1). The sample eligible for colorectal cancer screening comprised 43 647 patients with a mean (SD) age of 59.4 (7.5) years; 24 416 (55.9%) were women, 19 231 (44.1%) were men, 29 029 (66.5%) were white, and 9589 (22.0%) were black (Table 2).

**Table 1.** Sample Characteristics for Patients Eligible for Breast Cancer Screening^a^

Characteristic	Patients, No. (%)						
	2014-2015		2015-2016		2016-2017		Total (N = 26 269)
	Control (n = 8628)	Intervention (n = 1505)	Control (n = 6911)	Intervention (n = 1297)	Control (n = 6584)	Intervention (n = 1344)	
Age, mean (SD), y	60.7 (7)	61.1 (6.8)	60.1 (6.9)	60.8 (6.8)	60.1 (7.0)	60.9 (6.9)	60.4 (6.9)
Female sex	8628 (100.0)	1505 (100.0)	6911 (100.0)	1297 (100.0)	6584 (100.0)	1344 (100.0)	26 269 (100.0)
Race/ethnicity							
Non-Hispanic white	5521 (64.0)	728 (48.4)	4254 (61.6)	603 (46.5)	4083 (62.0)	684 (50.9)	15 873 (60.4)
Non-Hispanic black	2330 (27.0)	602 (40.0)	1982 (28.7)	536 (41.3)	1741 (26.4)	524 (39.0)	7715 (29.4)
Non-Hispanic Asian	200 (2.3)	66 (4.4)	179 (2.6)	58 (4.5)	183 (2.8)	54 (4.0)	740 (2.8)
Hispanic	135 (1.6)	12 (0.8)	124 (1.8)	18 (1.4)	126 (1.9)	14 (1.0)	429 (1.6)
Other	442 (5.1)	97 (6.4)	372 (5.4)	82 (6.3)	451 (6.8)	68 (5.1)	1512 (5.8)
Insurance							
Commercial	5364 (62.2)	869 (57.7)	4330 (62.7)	763 (58.8)	4142 (62.9)	720 (53.6)	16 188 (61.6)
Medicare	2753 (31.9)	483 (32.1)	2017 (29.2)	391 (30.1)	1952 (29.6)	464 (34.5)	8060 (30.7)
Medicaid	511 (5.9)	153 (10.2)	564 (8.2)	143 (11.0)	490 (7.4)	160 (11.9)	2021 (7.7)
Annual household income, $^b^							
<50 000	2627 (30.4)	663 (44.1)	2203 (31.9)	577 (44.5)	1960 (29.8)	612 (45.5)	8642 (32.9)
50 000-100 000	4720 (54.7)	531 (35.3)	3684 (53.3)	469 (36.2)	3644 (55.3)	441 (32.8)	13 489 (51.3)
>100 000	1191 (13.8)	301 (20.0)	945 (13.7)	235 (18.1)	924 (14.0)	275 (20.5)	3871 (14.7)
Missing	90 (1.0)	10 (0.7)	79 (1.1)	16 (1.2)	56 (0.9)	16 (1.2)	267 (1.0)
Charlson Comorbidity Index, median (IQR)	1 (0-2)	1 (0-3)	0 (0-2)	1 (0-2)	0 (0-2)	1 (0-3)	1 (0-2)

Abbreviation: IQR, interquartile range.

^a^ Data represent characteristics of patients who had a new or return visit with their primary care physician.

^b^ Annual household income was linked to each patient using United States Census data on median household income based on zip code.

**Table 2.** Sample Characteristics for Patients Eligible for Colorectal Cancer Screening^a^

Characteristic	Patients, No. (%)						
	2014-2015		2015-2016		2016-2017		Total (N = 43 647)
	Control (n = 17 231)	Intervention (n = 2534)	Control (n = 10 730)	Intervention (n = 1713)	Control (n = 9820)	Intervention (n = 1619)	
Age, mean (SD), y	60 (7.5)	60.3 (7.7)	58.8 (7.3)	59.3 (7.6)	58.7 (7.5)	59.5 (7.7)	59.4 (7.5)
Female sex	9693 (56.3)	1449 (57.2)	6004 (56)	944 (55.1)	5436 (55.4)	890 (55.0)	24 416 (55.9)
Race/ethnicity							
Non-Hispanic white	12 060 (70.0)	1457 (57.5)	7181 (66.9)	955 (55.8)	6482 (66)	894 (55.2)	29 029 (66.5)
Non-Hispanic black	3445 (20.0)	746 (29.4)	2303 (21.5)	515 (30.1)	2073 (21.1)	507 (31.3)	9589 (22.0)
Non-Hispanic Asian	465 (2.7)	120 (4.7)	305 (2.8)	75 (4.4)	301 (3.1)	62 (3.8)	1328 (3.0)
Hispanic	284 (1.6)	23 (0.9)	184 (1.7)	25 (1.5)	192 (2.0)	20 (1.2)	728 (1.7)
Other	977 (5.7)	188 (7.4)	757 (7.1)	143 (8.3)	772 (7.9)	136 (8.4)	2973 (6.8)
Insurance							
Commercial	11 282 (65.5)	1559 (61.5)	7451 (69.4)	1104 (64.4)	6823 (69.5)	992 (61.3)	29 211 (66.9)
Medicare	5064 (29.4)	767 (30.3)	2529 (23.6)	448 (26.2)	2359 (24.0)	442 (27.3)	11 609 (26.6)
Medicaid	885 (5.1)	208 (8.2)	750 (7.0)	161 (9.4)	638 (6.5)	185 (11.4)	2827 (6.5)
Annual household income, $^b^							
<50 000	4220 (24.5)	932 (36.8)	2710 (25.3)	617 (36.0)	2414 (24.6)	604 (37.3)	11 497 (26.3)
50 000-100 000	10 188 (59.1)	912 (36.0)	6111 (57.0)	649 (37.9)	5675 (57.8)	617 (38.1)	24 152 (55.3)
>100 000	2639 (15.3)	659 (26.0)	1775 (16.5)	426 (24.9)	1645 (16.8)	384 (23.7)	7528 (17.2)
Missing	184 (1.1)	31 (1.2)	134 (1.2)	21 (1.2)	86 (0.9)	14 (0.9)	470 (1.1)
Charlson Comorbidity Index, median (IQR)	0 (0-2)	1 (0-2)	0 (0-1)	1 (0-2)	0 (0-1)	1 (0-2)	0 (0-0)

Abbreviation: IQR, interquartile range.

^a^ Data represent characteristics of patients who had a new or return visit with their primary care physician.

^b^ Annual household income was linked to each patient using United States Census data on median household income based on zip code.

### Breast Cancer Screening

**Figure 1.**
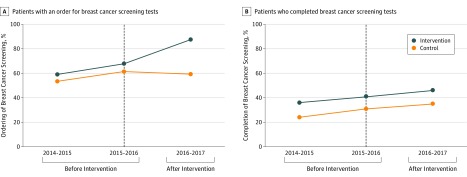
Breast Cancer Screening Rates by Practice Group and Year A, The percentage of patients who were eligible for breast cancer screening and left their primary care visit with an order for screening. B, The percentage of patients who had screening completed within 1 year after the visit. The active choice intervention was implemented at the intervention site during the period from 2016 to 2017. The vertical dashed black line separates the 2 preintervention years from the postintervention year.

Figure 1A displays the unadjusted rate of clinician ordering of breast cancer screening tests for the control and intervention practice groups by year. Clinician ordering of breast cancer screening tests was 53.5% in 2014-2015, 61.4% in 2015-2016, and 59.2% in 2016-2017 at control practices and 59.1% in 2014-2015, 68.8% in 2015-2016, and 87.5% in 2016-2017 at intervention practices. Adjusted preintervention trends during the first 2 years for clinician ordering of breast cancer screening tests did not differ between groups (adjusted odds ratio, 1.06; 95% CI, 0.85-1.33; *P* = .61). In adjusted analyses, there was a significant 22.2-percentage point increase (95% CI, 17.2-27.6 percentage points; *P* < .001) in clinician ordering of breast cancer screening tests for the intervention practices relative to control practices over time (Table 3).

**Table 3.** Adjusted Difference-in-Differences of Intervention Practices Relative to Control Practices Over Time

Screening	Adjusted Difference-in-Differences (95% CI), Percentage Points^a^^,^^b^	*P* Value
Breast cancer screening test		
Clinician ordering	22.2 (17.2 to 27.6)	<.001
Patient completion	0.1 (−4.0 to 4.3)	.45
Colorectal cancer screening test		
Clinician ordering	13.7 (8.0 to 18.9)	<.001
Patient completion	1.0 (−3.2 to 4.6)	.36

^a^ Adjusted difference-in-differences represent changes in intervention practices from the 2 preintervention years to the postintervention year relative to changes in control practices during the same time period.

^b^ Models are adjusted for patient demographics (age, sex, race/ethnicity, and household income), insurance, Charlson Comorbidity Index, clinic visit type (new or return), fixed effects by practice site, year, and calendar month.

Figure 1B displays the unadjusted rate of patient completion of breast cancer screening tests for the control and intervention practice groups by year. Patient completion of breast cancer screening was 24.1% in 2014-2015, 31.0% in 2015-2016, and 34.8% in 2016-2017 at control practices and 36.0% in 2014-2015, 40.8% in 2015-2016, and 45.9% in 2016-2017 at intervention practices. Adjusted preintervention trends during the first 2 years for patient completion of breast cancer screening tests did not differ between groups (adjusted odds ratio, 0.87; 95% CI, 0.72-1.06; *P* = .16). In adjusted analyses, there was not a significant difference (0.1 percentage points; 95% CI, −4.0 to 4.3 percentage points; *P* = .45) in patient completion of breast cancer screening tests for the intervention practices relative to control practices over time (Table 3). Unadjusted ordering and completion rates of breast cancer screening among patient subgroups by age, race/ethnicity, and income in the intervention and control practices before and after the intervention were similar to overall trends (eTables 1 and 2 in the Supplement).

### Colorectal Cancer Screening

**Figure 2.**
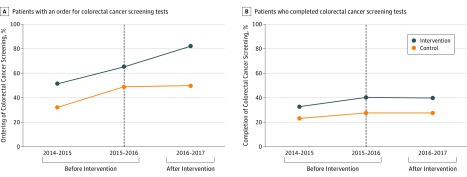
Colorectal Cancer Screening Rates by Practice Group and Year A, The percentage of patients who were eligible for colorectal cancer screening and left their primary care visit with an order for screening. B, The percentage of patients who had screening completed within 1 year after the visit. The active choice intervention was implemented at the intervention site during the period from 2016 to 2017. The vertical dashed black line separates the 2 preintervention years from the postintervention year.

Figure 2A displays the unadjusted rate of clinician ordering of colorectal cancer screening tests for the control and intervention practice groups by year. Clinician ordering of colorectal cancer screening tests was 32.1% in 2014-2015, 49.0% in 2015-2016, and 50.0% in 2016-2017 at control practices and 51.4% in 2014-2015, 65.2% in 2015-2016, and 82.0% in 2016-2017 at intervention practices. Adjusted preintervention trends during the first 2 years for clinician ordering of colorectal cancer screening tests did not differ between groups (adjusted odds ratio, 0.83; 95% CI, 0.66-1.05; *P* = .12). In adjusted analyses, there was a significant 13.7-percentage point increase (95% CI, 8.0-18.9 percentage points; *P* < .001) in clinician ordering of colorectal cancer screening tests for the intervention practices relative to control practices over time (Table 3).

Figure 2B displays the unadjusted rate of patient completion of colorectal cancer screening for the control and intervention practice groups by year. Individual colorectal cancer screening test rates were similar among both groups and, overall, were conducted by colonoscopy (88.6%), fecal occult blood test (6.8%), fecal immunochemical test (4.1%), sigmoidoscopy (0.3%), and multitargeted stool DNA test (0.3%). Patient completion of colorectal cancer screening tests was 23.1% in 2014-2015, 27.6% in 2015-2016, and 27.7% in 2016-2017 at control practices and 32.8% in 2014-2015, 40.4% in 2015-2016, and 39.7% in 2016-2017 at intervention practices. Adjusted preintervention trends during the first 2 years for clinician ordering of colorectal screening tests did not differ between groups (adjusted odds ratio, 1.10; 95% CI, 0.92-1.32, *P* = .27). In adjusted analyses, there was not a significant difference (1.0 percentage points; 95% CI, −3.2 to 4.6 percentage points; *P* = .36) in patient completion of colorectal cancer screening tests for the intervention practices relative to control practices over time (Table 3). Unadjusted ordering and completion rates of colorectal cancer screening tests among patient subgroups by age, race/ethnicity, income, and sex in the intervention and control practices before and after the intervention were similar to overall trends (eTables 3 and 4 in the Supplement).

## Discussion

Among a network of primary care practices, we found that an active choice intervention delivered to medical assistants through the EHR was associated with a significant 22-percentage point increase in clinician ordering of breast cancer screening tests and a significant 14-percentage point increase in clinician ordering of colorectal cancer screening tests, each relative to control practices over time. However, during a 1-year follow-up, the intervention was not associated with a significant change in patient completion of these screening tests. These findings demonstrate the potential of using nudges in the EHR to improve clinician decision-making but highlight that further interventions may need to be targeted to patients.

These findings expand our understanding of the association between practice environments and medical decision-making related to cancer screening. First, the magnitude of increase in clinician ordering was higher than in a previous pilot study in which the intervention was associated with a 12-percentage point increase for screening of both types of cancers.^11^ In the pilot study, the active choice intervention was targeted to both clinicians and medical assistants. Clinicians reported that this design led to alert fatigue and confusion in terms of which person was primarily responsible for placing the order for the screening tests. In this study, the intervention was directed only to medical assistants, who could create template orders for clinicians to review and prepare the patients by informing them that they were eligible for cancer screening and should discuss screening with their clinician. This design may have shifted some of the burden off clinicians and encouraged patients to prioritize a discussion on cancer screening.

Second, despite a large increase in clinician ordering of screening tests, the intervention was not associated with a significant change in patient completion of screening tests. This finding is in contrast to prior work using a similar intervention for influenza vaccination, which found that more than 99% of clinician orders resulted in patient vaccination.^13,17^ One important difference between influenza vaccination and cancer screening tests is the timing and amount of effort related to completing the order. Patients typically receive vaccinations before they leave their primary care visit, often directly from the clinician. Cancer screening tests involve a more burdensome and complex process. Patients often must schedule another appointment either with gastroenterology or radiology. Colonoscopy, the most common form of colorectal cancer screening in our study, requires bowel preparation and sedation. Patients in these primary care practices were mostly on their own to complete these steps and were not routinely sent reminders or given assistance to follow through with scheduling the tests and completing them. Future studies could evaluate ways to nudge scheduling and attendance at these appointments, as well as reduce the effort required to do so. For example, a randomized clinical trial in the same health system found that opt-out framing through mailed outreach led to a 3-fold increase in patient completion of colorectal cancer screening tests compared with an opt-in approach.^26^ Several other randomized clinical trials have found that direct outreach on colorectal cancer screening, to patients or their clinicians, also led to small increases in patient completion.^27,28^

### Limitations

This study is subject to some limitations. First, any observational study is susceptible to unmeasured confounders. However, the active choice intervention was evaluated using a difference-in-differences approach, which reduces potential bias from unmeasured cofounders by comparing changes in cancer screening over time between the intervention and control practices. Second, this study was conducted within a single health system, which may limit generalizability. However, we included 25 practice sites from 2 different states in urban and suburban clinics. Nonetheless, the findings should be confirmed in other settings. Third, we evaluated cancer screening eligibility and completion using data that were captured by 1 health system, and it is possible that some patients completed these tests at other locations. However, this would not bias the outcomes unless it was occurring differentially across groups other time.

## Conclusions

An active choice intervention in the EHR directed to medical assistants was associated with a significant increase in clinician ordering of breast and colorectal cancer screening tests. However, it was not associated with a significant change in patient completion of these tests during a 1-year follow-up. Our findings indicate that nudges facilitated by the EHR can increase clinician ordering of cancer screening tests but may need to be combined with other interventions to improve patient completion.

## Notes

Supplement. **eTable 1.** Unadjusted Ordering Rates for Breast Cancer Screening by Patient Subgroups in Intervention and Control Practices **eTable 2.** Unadjusted Completion Rates for Breast Cancer Screening by Patient Subgroups in Intervention and Control Practices **eTable 3.** Unadjusted Ordering Rates for Colorectal Cancer Screening by Patient Subgroups in Intervention and Control Practices **eTable 4.** Unadjusted Completion Rates for Colorectal Cancer Screening by Patient Subgroups in Intervention and Control Practices
