# Supplementary material for: Association of an Active Choice Intervention in the Electronic Health Record Directed to Medical Assistants With Clinician Ordering and Patient Completion of Breast and Colorectal Cancer Screening Tests
Source: JAMA Netw Open. 2019 Nov 15;2(11):e1915619. doi: 10.1001/jamanetworkopen.2019.15619 (PMC6902810; doi:10.1001/jamanetworkopen.2019.15619)
Supplement: Supplement. — eTable 1. Unadjusted Ordering Rates for Breast Cancer Screening by Patient Subgroups in Intervention and Control Practices eTable 2. Unadjusted Completion Rates for Breast Cancer Screening by Patient Subgroups in Intervention and Control Practices eTable 3. Unadjusted Ordering Rates for Colorectal Cancer Screening by Patient Subgroups in Intervention and Control Practices eTable 4. Unadjusted Completion Rates for Colorectal Cancer Screening by Patient Subgroups in Intervention and Control Practices [file jamanetwopen-2-e1915619-s001.pdf]

# Supplementary Online Content

Hsiang EY, Mehta SJ, Small DS, et al. Association of an active choice intervention in the electronic health record directed to medical assistants with clinician ordering and patient completion of breast and colorectal cancer screening tests. *JAMA Netw Open.* 2019;2(11):e1915619. doi:10.1001/jamanetworkopen.2019.15619

**eTable 1.** Unadjusted Ordering Rates for Breast Cancer Screening by Patient Subgroups in Intervention and Control Practices
**eTable 2.** Unadjusted Completion Rates for Breast Cancer Screening by Patient Subgroups in Intervention and Control Practices
**eTable 3.** Unadjusted Ordering Rates for Colorectal Cancer Screening by Patient Subgroups in Intervention and Control Practices
**eTable 4.** Unadjusted Completion Rates for Colorectal Cancer Screening by Patient Subgroups in Intervention and Control Practices

This supplementary material has been provided by the authors to give readers additional information about their work.

**eTable 1.** Unadjusted Ordering Rates for Breast Cancer Screening by Patient Subgroups in Intervention and Control Practices

| Breast Cancer - Ordering N, (%) | 2014-2015 (Pre) | 2015-2016 (Pre) | 2016-2017 (Post) |
|---|---|---|---|
| **Age 50-59** | | | |
| **Intervention** | 404/658 (61.4) | 417/598 (69.7) | 532/609 (87.4) |
| **Control** | 2384/4140 (57.6) | 2182/3499 (62.4) | 1971/3309 (59.6) |
| **Age ≥ 70** | | | |
| **Intervention** | 102/202 (50.5) | 100/164 (61.0) | 165/187 (88.2) |
| **Control** | 503/1210 (41.6) | 458/809 (56.6) | 447/813 (55.0) |
| **Race - White** | | | |
| **Intervention** | 349/728 (47.9) | 350/603 (58.0) | 559/684 (81.7) |
| **Control** | 2573/5521 (46.6) | 2324/4254 (54.6) | 2193/4083 (53.7) |
| **Race - Black** | | | |
| **Intervention** | 437/602 (72.6) | 418/536 (78.0) | 494/524 (94.3) |
| **Control** | 1608/2330 (69.0) | 1477/1982 (74.5) | 1245/1741 (71.5) |
| **Income - <$50,000** | | | |
| **Intervention** | 468/663 (70.6) | 449/577 (77.8) | 577/612 (94.3) |
| **Control** | 1766/2627 (67.2) | 1632/2203 (74.1) | 1406/1960 (71.7) |
| **Income - $50,000 to $100,000** | | | |
| **Intervention** | 294/531 (55.4) | 298/469 (63.5) | 369/441 (83.7) |
| **Control** | 2298/4720 (48.7) | 2089/3684 (56.7) | 2012/3644 (55.2) |
| **Income - >$100,000** | | | |
| **Intervention** | 125/301 (41.5) | 124/235 (52.8) | 218/275 (79.3) |
| **Control** | 518/1191 (43.5) | 488/945 (51.6) | 450/924 (48.7) |

© 2019 Hsiang EY et al. *JAMA Network Open.*

**eTable 2.** Unadjusted Completion Rates for Breast Cancer Screening by Patient Subgroups in Intervention and Control Practices

| Breast Cancer - Completion N, (%) | 2014-2015 (Pre) | 2015-2016 (Pre) | 2016-2017 (Post) |
|---|---|---|---|
| **Age 50-59** | | | |
| **Intervention** | 244/658 (37.1) | 232/598 (38.8) | 285/609 (46.8) |
| **Control** | 1046/4140 (25.3) | 1092/3499 (31.2) | 1093/3309 (33.0) |
| **Age 60-69** | | | |
| **Intervention** | 233/645 (36.1) | 231/535 (43.2) | 255/548 (46.5) |
| **Control** | 797/3278 (24.3) | 819/2603 (31.5) | 925/2462 (37.6) |
| **Age ≥ 70** | | | |
| **Intervention** | 65/202 (32.2) | 66/164 (40.2) | 77/187 (41.2) |
| **Control** | 240/1210 (19.8) | 230/809 (28.4) | 276/813 (33.9) |
| **Race - White** | | | |
| **Intervention** | 198/728 (27.2) | 213/603 (35.3) | 263/684 (38.5) |
| **Control** | 1048/5521 (19.0) | 1068/4254 (25.1) | 1264/4083 (31.0) |
| **Race - Black** | | | |
| **Intervention** | 274/602 (45.5) | 244/536 (45.5) | 285/524 (54.4) |
| **Control** | 811/2330 (34.8) | 816/1982 (41.2) | 732/1741 (42.0) |
| **Income - <$50,000** | | | |
| **Intervention** | 290/663 (43.7) | 250/577 (43.3) | 325/612 (53.1) |
| **Control** | 861/2627 (32.8) | 882/2203 (40.0) | 832/1960 (42.4) |
| **Income - $50,000 to $100,000** | | | |
| **Intervention** | 176/531 (33.1) | 189/469 (40.3) | 178/441 (40.4) |
| **Control** | 954/4720 (20.2) | 1002/3684 (27.2) | 1163/3644 (31.9) |
| **Income - >$100,000** | | | |
| **Intervention** | 74/301 (24.6) | 85/235 (36.2) | 107/275 (38.9) |
| **Control** | 253/1191 (21.2) | 246/945 (26.0) | 286/924 (31.0) |

© 2019 Hsiang EY et al. *JAMA Network Open.*

**eTable 3.** Unadjusted Ordering Rates for Colorectal Cancer Screening by Patient Subgroups in Intervention and Control Practices

| Colon Cancer - Ordering N, (%) | 2014-2015 (Pre) | 2015-2016 (Pre) | 2016-2017 (Post) |
|---|---|---|---|
| **Age 50-59** | | | |
| **Intervention** | 765/1213 (63.1) | 642/901 (71.3) | 692/829 (83.5) |
| **Control** | 3427/8851 (38.7) | 3280/6138 (53.4) | 2967/5586 (53.1) |
| **Age 60-69** | | | |
| **Intervention** | 435/926 (47.0) | 372/601 (61.9) | 479/572 (83.7) |
| **Control** | 1685/5870 (28.7) | 1577/3448 (45.7) | 1507/3150 (47.8) |
| **Age ≥ 70** | | | |
| **Intervention** | 102/395 (25.8) | 103/211 (48.8) | 157/218 (72.0) |
| **Control** | 419/2510 (16.7) | 397/1144 (34.7) | 433/1084 (39.9) |
| **Race - White** | | | |
| **Intervention** | 720/1457 (49.4) | 627/955 (65.7) | 733/894 (82.0) |
| **Control** | 3364/12060 (27.9) | 3227/7181 (44.9) | 3041/6482 (46.9) |
| **Race - Black** | | | |
| **Intervention** | 400/746 (53.6) | 324/515 (62.9) | 421/507 (83.0) |
| **Control** | 1583/3445 (46.0) | 1414/2303 (61.4) | 1232/2073 (59.4) |
| **Income - <$50,000** | | | |
| **Intervention** | 484/932 (51.9) | 385/617 (62.4) | 497/604 (82.3) |
| **Control** | 1905/4220 (45.1) | 1680/2710 (62.0) | 1481/2414 (61.4) |
| **Income - $50,000 to $100,000** | | | |
| **Intervention** | 464/912 (50.9) | 431/649 (66.4) | 503/617 (81.5) |
| **Control** | 2758/10188 (27.1) | 2648/6111 (43.3) | 2573/5675 (45.3) |
| **Income - >$100,000** | | | |
| **Intervention** | 340/659 (51.6) | 291/426 (68.3) | 319/384 (83.1) |
| **Control** | 818/2639 (31.0) | 869/1775 (49.0) | 816/1645 (49.6) |
| **Gender Male** | | | |
| **Intervention** | 560/1085 (51.6) | 490/769 (63.7) | 604/729 (82.9) |
| **Control** | 2318/7538 (30.8) | 2212/4726 (46.8) | 2153/4384 (49.1) |
| **Gender Female** | | | |
| **Intervention** | 742/1449 (51.2) | 627/944 (66.4) | 724/890 (81.3) |
| **Control** | 3213/9693 (33.1) | 3042/6004 (50.7) | 2754/5436 (50.7) |

© 2019 Hsiang EY et al. *JAMA Network Open.*

**eTable 4.** Unadjusted Completion Rates for Colorectal Cancer Screening by Patient Subgroups in Intervention and Control Practices

| Colon Cancer - Completion N, (%) | 2014-2015 (Pre) | 2015-2016 (Pre) | 2016-2017 (Post) |
|---|---|---|---|
| **Age 50-59** | | | |
| **Intervention** | 443/1213 (36.5) | 373/901 (41.4) | 329/829 (39.7) |
| **Control** | 2253/8851 (25.5) | 1724/6138 (28.1) | 1526/5586 (27.3) |
| **Age ≥ 70** is below; **Age 60-69** | | | |
| **Intervention** | 290/926 (31.3) | 243/601 (40.4) | 240/572 (42.0) |
| **Control** | 1321/5870 (22.5) | 976/3448 (28.3) | 938/3150 (29.8) |
| **Age ≥ 70** | | | |
| **Intervention** | 97/395 (24.6) | 76/211 (36.0) | 74/218 (33.9) |
| **Control** | 404/2510 (16.1) | 266/1144 (23.3) | 260/1084 (24.0) |
| **Race - White** | | | |
| **Intervention** | 495/1457 (34.0) | 417/955 (43.7) | 367/894 (41.1) |
| **Control** | 2617/12060 (21.7) | 1875/7181 (26.1) | 1742/6482 (26.9) |
| **Race - Black** | | | |
| **Intervention** | 233/746 (31.2) | 187/515 (36.3) | 193/507 (38.1) |
| **Control** | 979/3445 (28.4) | 758/2303 (32.9) | 665/2073 (32.1) |
| **Income - <$50,000** | | | |
| **Intervention** | 289/932 (31.0) | 224/617 (36.3) | 224/604 (37.1) |
| **Control** | 1158/4220 (27.4) | 855/2710 (31.5) | 749/2414 (31.0) |
| **Income - $50,000 to $100,000** | | | |
| **Intervention** | 279/912 (30.6) | 262/649 (40.4) | 237/617 (38.4) |
| **Control** | 2181/10188 (21.4) | 1614/6111 (26.4) | 1536/5675 (27.1) |
| **Income - >$100,000** | | | |
| **Intervention** | 252/659 (38.2) | 202/426 (47.4) | 177/384 (46.1) |
| **Control** | 608/2639 (23.0) | 467/1775 (26.3) | 424/1645 (25.8) |
| **Gender Male** | | | |
| **Intervention** | 371/1085 (34.2) | 325/769 (42.3) | 294/729 (40.3) |
| **Control** | 1756/7538 (23.3) | 1329/4726 (28.1) | 1282/4384 (29.2) |
| **Gender Female** | | | |
| **Intervention** | 459/1449 (31.7) | 367/944 (38.9) | 349/890 (39.2) |
| **Control** | 2222/9693 (22.9) | 1637/6004 (27.3) | 1442/5436 (26.5) |

© 2019 Hsiang EY et al. *JAMA Network Open.*
